# How can we improve embryo production and pregnancy outcomes of Holstein embryos produced *in vitro*? (12 years of practical results at a California dairy farm)

**DOI:** 10.1590/1984-3143-AR2020-0053

**Published:** 2020-08-14

**Authors:** Daniela Garcia Borges Demetrio, Eduardo Benedetti, Clarice Garcia Borges Demetrio, Julio Fonseca, Mayara Oliveira, Alvaro Magalhaes, Ricarda Maria dos Santos

**Affiliations:** 1 RuAnn Genetics, Riverdale, CA, United States; 2 Arizona Dairy Co, Mesa, AZ, United States; 3 Escola Superior de Agricultura “Luiz de Queiroz”, Universidade do Estado de São Paulo, Piracicaba, SP, Brasil; 4 Faculdade de Medicina Veterinária, Universidade Federal de Uberlândia, Uberlândia, MG, Brasil

**Keywords:** embryo, Holstein, *in vitro* production, pregnancy, recipient

## Abstract

Genomic evaluations have revolutionized dairy cattle breeding, and the demand for embryos produced from very young heifers with high genetic merit has increased over time. The combination of low oocyte recovery, young age of donors, and milk production status can make the *in vitro* embryo production (IVP) of Holstein cattle incredibly challenging. Several factors need to be coordinated to obtain a live calf from an IVP embryo, but the quality of the oocyte at the start of the process is one of the key factors. Aspects related to oocyte quality, laboratory quality control, embryo quality and recipient selection are addressed here, based on the measures that the RuAnn Genetics Laboratory (Riverdale, California, USA) adopted in the last 12 years, with the goal of improving production of live, healthy calves from Holstein embryos. Follicular wave synchronization and stimulation with follicular stimulating hormone (FSH) is necessary to improve oocyte quality and consequently embryo production. Laboratory quality control and the use of high-quality supplies are essential to reduce variability in production and facilitate identification of other factors that might interfere with embryo production. High pregnancy rates can be achieved with good quality embryos selected at optimal time and stage of development, transferred by an experienced embryo transfer (ET) technician, to well managed recipients 7 or 8 days after estrus. Attention to detail at every step of the process is crucial to success.

## Introduction

Embryo production has proven to be a powerful technology for genetic improvement of dairy animals, primarily to propagate the genes of females with superior genetic values and lineage. According to data collected by the American Embryo Transfer Association (AETA) Statistical Committee in 2018 (Demetrio and Wehrman, 2019), dairy cows produce an average of 15.7 oocytes and 3.3 viable embryos per ovum pick up (OPU) session. Dairy cattle breeding and selection have been revolutionized by the use of genomic selection, and the demand for embryos produced from very young heifers (<10 months) with high genetic merit increased overtime. Lactating donor cows can have decreased oocyte quality, lower fertilization rates, and impaired early embryonic development due to their lactational metabolic challenges (Leroy et al., 2011), and higher incidence of postpartum metabolic and infectious diseases (Ribeiro et al., 2016). The combination of low oocyte recovery, young age of donors, and milk production status can make the IVP of Holstein cattle incredibly challenging.

Several factors need to be coordinated to obtain a live calf from an IVP embryo. The donor must provide a good quality oocyte, that can be matured and be successfully fertilized. The embryo needs to develop *in vitro* until day 7 post fertilization and be carefully selected, loaded, and transferred by a qualified technician. Semen, laboratory equipment, IVP media, quality control protocols and laboratory technicians will determine the success of the process. The care taken before and after the embryo is produced will determine pregnancy outcome (Sirard, 2018; Demetrio, 2019; Hansen, 2020). The goal is to have a pregnant recipient that can successfully carry a healthy calf to term.

Over the past decade, the success of commercial IVP has significantly improved, as higher blastocyst rates, better cryotolerance, higher pregnancy rates, lower pregnancy loss and decreased incidence of large offspring syndrome have been reported. Nevertheless, embryos generated *in vitro* still differ from their *in vivo* produced counterparts (Hansen, 2020). Approximately 80-90% of immature bovine oocytes undergo nuclear maturation *in vitro*, about 80% undergo fertilization, 30-40% develop to blastocyst stage, and around 50% of the transferred embryos establish a pregnancy (Wrenzycki, 2016).

**Figure 1:**
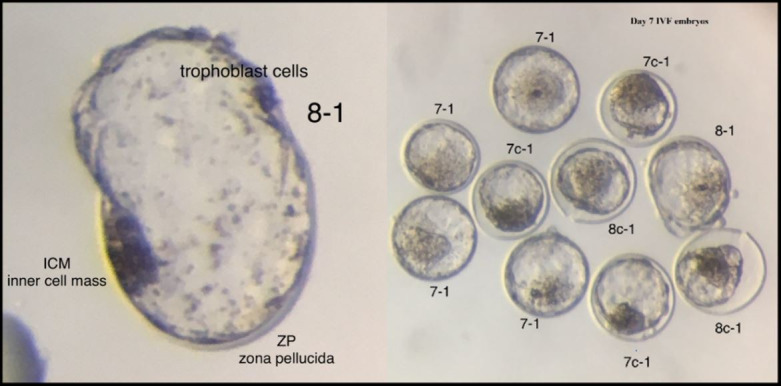
Grade 1 Day 7 IVP Embryos. The first number refers to the stage, 7 (expanded blastocyst), 8 (hatching blastocyst) and c (collapsed), and the second number refers to the grade (ICM – inner cell mass, ZP – zona pellucida).

RuAnn genetics (Riverdale, California, USA) produced and transferred over 32,503 *in vivo* derived Holstein embryos in the last 11 years with 63% pregnancy rate in virgin heifer recipients (n=23,488 ET) and 51% in lactating cow recipients (n=9,015) (Demetrio et al., 2019). Since we established the in house IVP laboratory at the dairy in 2008, our main goal was to make *in vivo*-like IVP embryos, with similar pregnancy outcomes (Figure 1).

This manuscript will describe the measures that the RuAnn Genetics Laboratory adopted to improve success of IVP systems in Holsteins. Conclusions are based on results obtained in the last 12 years and will focus on oocyte quality, laboratory quality control, embryo quality and recipient selection.

## Data collection and protocols

Our data were collected frequently to monitor performance and improve results. It was collected during the routine in the farm without an experimental design and analyzed retrospectively. The protocols described herein were developed over a 12-year period of continual refinement of techniques to improve embryo production and pregnancy results.

RuAnn Genetics, located in Riverdale, San Joaquin Valley, California, USA (N36°28’26.945”, W119°55’47.65”), milks 4,500 Holstein cows at RuAnn and Maddox Dairies, with a 305-d mature-equivalent milk production of 12,800 kg. Embryo production and transfer have been a part of the genetic program since 1985. OPU, *in vitro* maturation (IVM), fertilization (IVF), culture (IVC), ET and freezing for direct transfer have been performed in house since the establishment of the in-house laboratory in 2008. Between 6,000 to 10,000 embryos are produced per year. In 2019, 6,985 *in vitro* (85%) and *in vivo* (15%) embryos were produced, 4,348 embryos (62%) were used for Maddox Dairy (transferred or exported) and 2,637 were produced for other dairy or beef farmers (transferred fresh or cryopreserved).

Holstein heifers (ranging in age and physiological status of 8 months old to 4 months pregnant), lactating cows and dry cows were used as oocyte donors. Cows with very good or excellent classification scores (Holstein Association linear classification), with good family and production records, and 3 or more complete lactations, were dried up to become permanent donors. Approximately 50% of the donors were virgin heifers. All donors were synchronized with gonadorelin (GnRH, Fertagyl^®^, Merck Animal Health, USA) and stimulated with FSH (Folltropin^®^, Vetoquinol, USA) prior to OPU and were not selected by quantity of oocytes, but by the goal of multiplying desired genetics. GnRH (129 µg, IM) was given twice, one week apart, to induce CL formation and increase plasma concentrations of progesterone, and to enhance synchronization of follicular recruitment. The first injection of FSH was administered 36 hours after the second GnRH injection. A total dosage of 245 IU of FSH for lactating cows and 175 IU for heifers and dry cows was divided into 5 IM injections. OPU was performed 16 to 20 hours after the last FSH injection for heifers or 26 to 30 hours for cows (Table 1).

**Table 1:** Protocol using GnRH and FSH to prepare donor cows and heifers for OPU.

***WEEK***	***TIME***	***COW SCHEDULE***						
		**Sun**	**Mon**	**Tues**	**Wed**	**Thu**	**Fri**	**Sat**
1	7:00 AM							
	4:00 PM						GnRH	
2	7:00 AM							
	4:00 PM						GnRH	
3	7:00 AM	FSH	FSH	FSH	**OPU**			
	4:00 PM	FSH	FSH					
								
**WEEK**	**TIME**	**HEIFER SCHEDULE**						
		**Sun**	**Mon**	**Tues**	**Wed**	**Thu**	**Fri**	**Sat**
1	7:00 AM							GnRH
	4:00 PM							
2	7:00 AM							GnRH
	4:00 PM							
3	7:00 AM		FSH	FSH	**OPU**			
	4:00 PM	FSH	FSH	FSH				

Each Monday and Wednesday, donors were brought to the laboratory, properly restrained, and received an epidural anesthesia with lidocaine 2% solution prior to OPU. Cumulus oocytes complexes (COCs) were retrieved using a medical ultrasound equipped with a 6.5 MHz micro-convex transvaginal transductor (Mindray DP50, China) adapted for bovine ovary aspiration. A 2 mm Teflon tubing system was attached to an oocyte aspiration dish with grid (SPI^TM^, USA) and the needle. A negative pressure produced by a vacuum pump (COOK^®^, Australia) was applied to the filter at 20-30 mL/minute flow rate. All visible follicles (>3 mm) were aspirated using a 20G disposable hypodermic needle. Modified Dulbecco’s phosphate buffered saline (DPBS, ABT Complete Flush, ABT 360^®^, USA) with 10 IU/mL heparin was used to flush the system to avoid coagulation. The oocyte aspiration dish and flush medium were kept at 37 °C. After the aspiration was performed, the dish containing the follicular fluid was rinsed with modified DPBS until the fluid got clear and could be searched under a stereomicroscope. We adopted the use of aspiration oocyte dishes to recover COCs instead of a 50 mL tube in 2015. Doing so reduced two steps of the process, since OPU and searching occur using the same plate, thereby reducing oocyte manipulation. One noticeable result has been an increase in the number of layers of cumulus cells on recovered COCs. The retrieved COCs were placed in a 6-well dish with 1000µL of wash medium (TCM 199 medium supplemented with HEPES) and washed at least 3 times. This process was timed and did not take more than 15 minutes.

**Figure 2:**
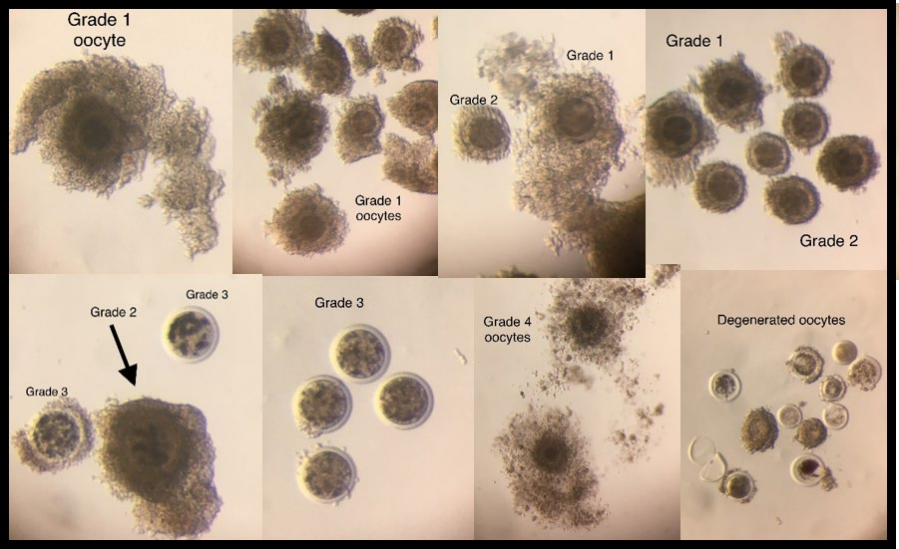
Classification of COCs used at RuAnn Genetics. Grade 1 COCs have more than 5 compact cumulus cells layers covering all of the zona pelucida (ZP) surface. The ooplasm is dense, homogeneous, and non-pigmented. Grade 2 COCs have more than 3 compact cumulus cells layers covering all or at least 80% of the ZP surface. The ooplasm is dense, homogeneous, and non-pigmented. Grade 1 oocytes with irregular, pigmented ooplasm are downgraded to grade 2. Grade 3 COCs have fewer than 3 compact cumulus cells layers and the ooplasm may be homogenous or not. Grade 2 oocytes with irregular, pigmented ooplasm are downgraded to grade 3. Grade 4 COCs have expanded cumulus cells layers. If the ooplasm is retracted, the oocyte will be considered degenerated. Oocytes with retracted or noticeably light and/or irregular ooplasm, cracked and empty ZP, or smaller sized or primary oocytes, are considered degenerated.

The recovered COCs were classified as grade 1 to 4 (viable) or degenerated based on our embryo production results and the classification described by Antunes et al. (2008) and Kanagawa et al. (1995). It takes into consideration the number and presentation of cumulus cell layers and the ooplasm homogeneity (Figure 2). The degenerated oocytes were not included with the viable oocytes for IVM but were accounted for to determine the oocyte recovery rate.

The COCs underwent IVM for 18 to 24 hours to become competent for fertilization. The IVM was conducted in tubes inside a portable incubator (38.5 °C) or in 4-well dishes with supplemented TCM199, inside an incubator (38.7 °C, 5% CO_2_ in a humidified atmosphere) with a maximum of 40 COCs per tube or drop. The matured COCs were removed from the IVM tube or dish, washed once in IVF medium (supplemented TALP), and moved to the fertilization dish (a drop of 50µL medium overlaid with 900µL oil at 38.7 °C, 5% CO_2_ in a humidified atmosphere, in 4-well dishes). A straw of semen was thawed, and its contents were placed on top of two layers (40 and 80%) of silica-based colloidal medium (Puresperm^®^, Nidacon International, USA) and centrifuged to separate the live spermatozoa from the cryoprotectant, seminal plasma and dead spermatozoa. The supernatant was removed, and the remaining spermatozoa were washed with IVF media and centrifuged once again. The motility and concentration on the final pellet were evaluated by the i-Sperm^®^ (Aidmics Biotechnology Co., LTB, Taiwan) device to determine the final insemination volume. Female sex sorted semen was used in 90% of our procedures. The oocytes were usually inseminated with 10 to 25µL of the spermatozoa solution to achieve a final concentration of 2 million sperm/mL. The IVF underwent for 7 to 12 hours. After fertilization, the cumulus cells surrounding the zygotes were removed by pipetting (also known as stripping). Zygotes were washed and placed into droplets of culture medium [70µL drop of supplemented synthetic oviduct fluid (SOF) medium overlaid with 900µL oil at 38.7 °C, 6% CO_2_ and 5% O_2_ in a humidified atmosphere]. Some of the grade 3 and 4 oocytes placed in maturation initially were shrunken or degenerated and were removed from culture. There was a 7% reduction in the number of oocytes from IVM to IVC. On day 3 of culture, morulae with 8-16 cells should be present and the cleavage rate was calculated (total cleaved divided by the total oocytes in IVC). Unfertilized oocytes and zygotes with less than 4 cells were removed from the drop. The medium from the drop was removed (65µL) and replaced by a new IVC-D3 medium containing different nutrients necessary for this phase. On day 5 of culture, compact morulae and early blastocysts should be present and an estimate of the final embryo production was made. The medium from the drop was removed once more (65µL) and replaced by a new medium (IVC-D5) containing different nutrients necessary for this phase. On day 6 of culture, compact morulae and blastocysts should be present. Day 6 blastocysts were removed from culture for transfer depending on recipient availability. On day 7 of culture, the remaining embryos were transferred fresh to recipients or cryopreserved for direct transfer (15% of the viable embryos). A good quality day 7 IVF expanded, or hatched blastocyst should have a defined inner cell mass and clear trophoblast cells (Figure 1). Manipulation of day 6 and 7 embryos was done at 36.5 °C in holding media (HEPES-SOF) and did not take longer than 10 minutes, to avoid medium pH changes that can damage the embryo. The embryos that did not reach the early blastocyst stage on Day 7 were discarded. All the embryos were placed in a portable straw incubator at 35 °C to 36.5 °C. The time from loading the first embryo to ET of the last embryo did not exceed 7 hours. Embryo production rates were calculated by the number of embryos produced divided by the number of oocytes in IVC. We also divided the number of embryos by the sum of grade 1 and 2 oocytes recovered, and used it as a quality control check, expecting an embryo production rate higher than 60%. Only embryos that were transferred fresh or cryopreserved were included in this calculation.

Summers at the San Joaquin Valley are hot and dry, and winters are cool and wet. During heat waves temperatures may rise up to 46 °C. June, July, August, September, and October, when the first artificial insemination conception rate dropped below 40%, were called critical months.

Natural heats from virgin Holstein heifers between 13.5 to 15.5 months old (first and second services only) were observed daily. First or second lactation, first service, Holstein lactating cows over 80 days in milk synchronized with Presynch-Ovsynch protocol, were also used as recipients (available Thursdays and Fridays). Embryo transfer was performed 6 to 9 days after estrus, preferably 7 and 8, depending on recipient availability. Only recipients that had a corpus luteum (CL) on the day of transfer received an embryo.

Pregnancy diagnosis was performed in heifers by transrectal ultrasound examination at 32 to 46 days post estrus, and in lactating cows by the detection of pregnancy-associated glycoproteins in serum (IDEXX, USA) at 30 days post estrus and reconfirmed at 80 days by transrectal ultrasound examination. Pregnancy rate was calculated by the number of pregnant recipients divided by the total ET.

## Data analysis

The standard model used to analyze counts over time or space is the Poisson regression model. For this very simple model we assume that events happen independently, singly, and at random at some constant underlying rate. However, our oocyte and embryo count are overdispersed, and the variability of the data is larger than the variability specified by the Poisson model (Hinde and Demétrio, 1998). The response variables number of oocytes and number of embryos have two extra-variability sources, one caused by the heterogeneity of the animals and the other by the random effects of donor and sire. A mixed negative binomial model (Molenberghs et al., 2007) was used to analyze the data taking into consideration both factors. Alternative nested models with different combinations of the covariates in the linear predictor were compared using likelihood ratio tests. For an individual binary outcome as is the pregnancy variable (with only two possible outcomes: pregnant or not) the basic probability model is the Bernoulli distribution (Collett, 2002). Alternative nested models, with different combinations of the covariates in the linear predictor of the logistic regression model, were compared using likelihood ratio tests. All the models were fitted using R (R Core Team, 2019).

## Oocyte quality

Culture conditions throughout IVP may have influence on the developmental potential of the early embryo, but the quality of the oocyte at the start of the process is the key factor determining the proportion of oocytes developing to the blastocyst stage (Lonergan and Fair, 2016). According to the data collected by the AETA Statistical Committee in 2017 and 2018 (Table 2), more oocytes per OPU were recovered from beef breeds than dairy breeds, and both produced more viable embryos per OPU when FSH was used to stimulate the donor cows (Demetrio and Wehrman, 2018, 2019).

**Table 2:** Comparison of dairy and beef donors for performance of IVP based on 2017 and 2018 data from the American Embryo Transfer Association Statistical Committee.

**2017**	***DAIRY***			***BEEF***		
	***NO FSH***	***FSH***	***TOTAL***	***NO FSH***	***FSH***	***TOTAL***
Total OPUs	46,847	32,640	79,487	3,302	20,138	23,440
Oocytes per OPU	15.6	16.6	16.0	17.8	22.5	21.8
Total Viable Embryos	107,330	170,980	278,310	16,022	126,805	142,827
Viable Embryos per OPU	2.3	5.2	3.5	4.9	6.3	6.1
% Viable Embryos (Viable/Recovered)	18%	31%	24%	31%	29%	29%
***2018***	**DAIRY**			**BEEF**		
	***NO FSH***	***FSH***	***TOTAL***	***NO FSH***	***FSH***	***TOTAL***
Total OPUs	54,515	30,290	84,805	3,789	20,233	24,022
Oocytes per OPU	15.4	16.3	15.7	20.6	22.4	22.1
Total Viable Embryos	117,237	163,647	280,884	20,675	144,469	165,144
Viable Embryos per OPU	2.2	5.4	3.3	5.5	7.1	6.9
% Viable Embryos (Viable/Recovered)	14%	33%	21%	26%	32%	31%

In 2017, we decided to synchronize and stimulate 100% of our donors with FSH and we observed an increase of almost 2 embryos per donor per OPU. Since we start with fewer oocytes when working with Holsteins, our goal was to obtain a larger proportion of grade 1 and 2 oocytes per OPU procedure to increase our embryo production per cow. According to our data (Table 3), we have embryo production rates higher than 60% from the combination of grade 1 and 2 oocytes. We have tried different protocols during the last several years. The one we have been using recently is illustrated on Table 1. Due to the almost prohibitive cost of FSH, we adopted protocols using lower dosages. Recovery rates dropped when the time from the last FSH to OPU exceeded 30 hours, especially for heifers. Table 3 summarizes the data from 3,233 Holstein OPUs (RuAnn donors only) performed in our laboratory from January 2017 to March 2020. The donor cows were divided into one of four groups: heifers younger than 10 months, heifers older than 10 months, lactating cows, and dry cows. We also divided them in 3 groups based on oocyte production: less than 10 oocytes (low), 11 to 20 oocytes (medium) and more than 20 oocytes (high).

**Table 3:** Oocyte recovery and embryo production from Holstein OPUs performed at RuAnn donors from January 2017 to March 2020.

**Donor Group/Quantity of Oocytes**	**OPUs**	**Total Oocytes per OPU**	**IVC Oocytes per OPU**	**Grade 1 and 2 Oocytes/** **Total Oocytes**	**Cleavage Rates**	**Embryos per Donor**	**Embryos/IVC Oocytes**	**Embryos/** **Grade 1 and 2 Oocytes**
**> 20 oocytes per OPU**	**517**	**34.1**	**29.3**	**35%**	**78%**	**7.7**	**26%**	**64%**
<10mo Heifers	65	34.3	28.0	33%	69%	4.8	17%	42%
>10mo Heifers	77	29.4	26.2	41%	76%	7.0	27%	57%
Lactating Cows	117	32.1	27.5	30%	77%	6.7	24%	70%
Dry Cows	258	36.4	31.3	37%	81%	9.1	29%	68%
**> 10-20 oocytes per OPU**	**1192**	**17.0**	**14.5**	**35%**	**78%**	**4.1**	**28%**	**69%**
<10mo Heifers	157	16.5	14.3	34%	74%	3.2	22%	56%
>10mo Heifers	385	16.2	14.2	39%	81%	4.6	32%	71%
Lactating Cows	257	17.3	14.7	27%	77%	3.5	24%	77%
Dry Cows	393	17.9	14.9	37%	79%	4.5	30%	68%
**<10 oocytes per OPU**	**1524**	**8.2**	**6.3**	**31%**	**78%**	**1.7**	**27%**	**67%**
<10mo Heifers	177	8.2	6.5	30%	76%	1.5	23%	59%
>10mo Heifers	710	7.9	6.2	34%	80%	1.9	30%	71%
Lactating Cows	371	8.0	6.3	23%	74%	1.5	23%	77%
Dry Cows	266	9.0	6.5	35%	79%	1.7	26%	55%
**TOTAL**	**3233**	**15.6**	**13.0**	**34%**	**78%**	**3.6**	**27%**	**67%**
<10mo Heifers	399	15.7	13.1	33%	73%	2.7	21%	52%
>10mo Heifers	1172	12.0	10.2	37%	80%	3.1	30%	69%
Lactating Cows	745	15.0	12.5	27%	76%	3.0	24%	74%
Dry Cows	917	20.5	17.1	36%	80%	5.0	29%	66%

**Figure 3:**
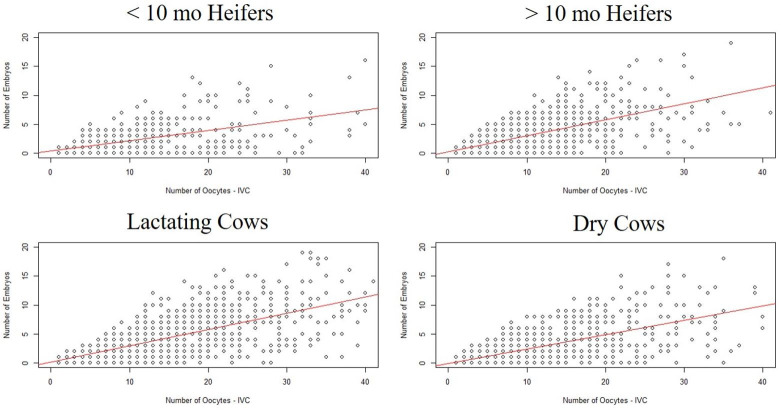
Dispersion plot of number of embryos versus number of oocytes on IVC.

**Figure 4:**
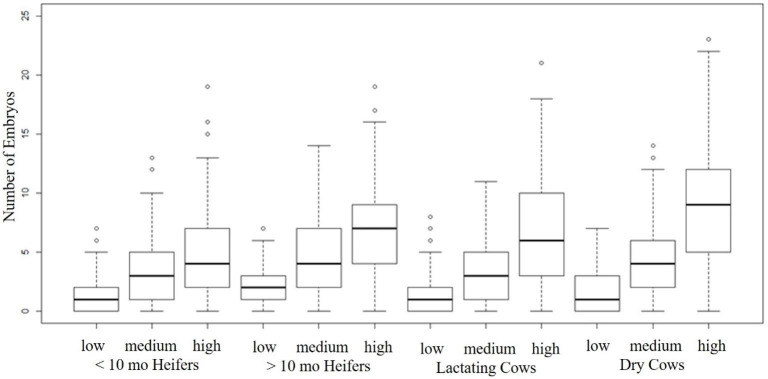
Boxplot of number of embryos by donor group and by oocyte production group.

A dispersion plot of the number of embryos per donor by the number of oocytes per donor (Figure 3) and a boxplot of the number of embryos by donor group and by oocyte production group (Figure 4) indicate that there is great variability in both variables among donor cows. Higher embryo production will be achieved by donors that start with a higher number of oocytes. When we fit a mixed negative binomial model to the number of oocytes with donor dam group as fixed effect, and donor dam as random effect, there is evidence of a significant effect of donor and donor group on the total number of oocytes. A higher number of total oocytes was recovered from dry cows when compared to the other donor groups. When we fit a mixed negative binomial model with donor group, oocyte production group and interaction as fixed effects, and donor and sire as random effects to the number of embryos, there is significant evidence of all effects except for the interaction. The number of embryos produced depend on the donor and sires. Dry donors produce more embryos than heifers and lactating cows, probably because they do not have any metabolic challenges while the lactating cows are producing milk and the heifers are still growing. The number of oocytes recovered from older heifers is inferior than younger heifers. We start to OPU the genetically superior genomic tested heifers at 8 months of age and some will be aspirated every 3 to 4 weeks until 100 days of pregnancy. Most of those heifers that are worked frequently, start with high number of oocytes but the oocyte number decreases overtime. Despite the higher number of oocytes produced by the younger heifers, the embryo production rate is lower than the older heifers (21% vs 30%).

As mentioned before, we do not select our donor cows by the number of oocytes, but if we did, we would increase the average of embryos per cow and reduce the cost of embryo production. As long as we do not mind foregoing producing offspring from highest genetic merit cows, of course!

## Laboratory quality control and assurance

The impact of culture media formulations on mammalian embryo development has been extensively documented over the preceding five decades. Improvements in the success of IVP can be attributed, in no small way, to the continued efforts of laboratories around the world to optimize media formulations. However, what is evident is that media are but one aspect of the embryo culture system (Gardner and Lane, 2003). To support the development of a viable and healthy embryo capable of resulting in a healthy offspring, one needs to look far beyond the formulation of the culture media employed. There is an absolute necessity for an effective quality management and assurance system to ensure that variables such as equipment, laboratory air quality, oil overlay, and lot numbers of consumables do not negatively impact embryo health (Gardner and Kelley, 2017). With the intention to avoid chemical and physical stress factors that might affect the oocyte and embryo, we adopted several measures based on information interchanged with other IVP bovine and human practitioners, research papers, and most of all, trial and error. It is impossible to isolate each factor and how it affected the data specifically, but we will describe some of the changes that had an impact on our results.

The IVP laboratory is a complex system and involves many activities and people. Therefore, the system’s complexity requires that many processes and procedures be performed correctly. Thus, the establishment of a Standard Operating Procedure (SOP) is an important part of a successful quality system, as it allows maintaining consistency and decreasing the chances of error during the execution of procedures. Another important point is to keep written records of all changes that have been made in the laboratory, such as: dates of changes of batches of media and oil, cleaning and gas exchange of the incubators and any other minor or major changes in the procedure that could affect the embryo production. This facilitates the possibility of identifying any error in the process.

Incubators in the IVF laboratory play a pivotal role in providing a stable and appropriate culture environment required for optimizing embryo development outcome. In 2015, we started to perform IVC under low oxygen (5%). We had only one large incubator and the amount of nitrogen required to operate was extremely high because of the need to open the door often. In 2016, we purchased a benchtop incubator with 2 different chambers. The combination of low oxygen levels with rapid recovery times for atmosphere and temperature improved the quality of our embryos. It also became more cost effective.

We always believed in the use of a sequential media for IVC because different nutrients are supplied at different stages of development based on the embryo’s requirements. In 2015, we reduced the amount of fetal calf serum and in 2018, it was entirely eliminated from culture medium. The goal was to prepare defined medium to minimize variation of the unknown components that occurs naturally in serum. Embryo quality and survival to cryopreservation consequently improved.

The perfect oil for IVP should do nothing but protect the culture medium from evaporation. It should not have any negative influence on the gametes, embryos, or medium by adding unwanted or removing essential components. We have had a number of issues with different batches of oil in the laboratory, and in 2016 we decided to purchase oil used in human IVP (Ovoil^TM^, Vitrolife, USA) and the variability in our embryo production results decreased dramatically.

One important factor that can have a detrimental impact on the IVF treatment outcome, but is sometimes overlooked, is the quality of the plastics used. It is also worth considering the fact that the effects of suboptimal utensils are cumulative; the negative impact will increase if more than one toxic item is used. We purchase IVP certified utensils (dishes, tips, ET straws, etc.) wherever possible, that have lower risk of toxic molecules from the plastic entering the medium or oil.

High quality supplies and precise equipment have become essential throughout our IVP process, enabling the necessary laboratory stability which allows us to be more accurate when measuring other critical aspects of production such as individual donor performance, mating interactions and others. Overall production costs increase, but it is usually compensated for higher yield of blastocysts and pregnancies.

## Embryo quality and recipient selection

**Figure 5:**
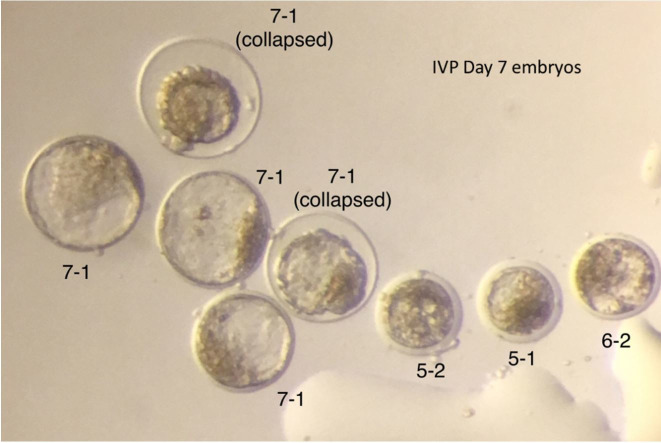
A group of IVP Day 7 embryos with expanded blastocysts (7) and some that are behind in development (5 and 6) and disorganized. The first number refers to the stage and the second number refers to the grade.

Morphological evaluation has been the method of embryo selection for many years and remains the primary approach of embryo assessment. However, this evaluation method poses limitations, not only arising from the subjectivity of the embryologist, but also because of the evaluation system itself, which views embryo development statically (Gardner, 2015). Currently, time-lapse technology is being used in combination with analysis of the metabolism and gene expression for analyzing the developmental potential and viability of mammalian embryos (Gardner and Kelley, 2017). Unfortunately, we do not have an expensive time-lapse incubator in our laboratory, but we have incorporated the observation of the embryo during different phases as another way to determine embryo viability. Based on our observations and data, embryos that reach compact morula stage on day 5 of IVC, blastocyst stage on day 6, and expanded blastocyst or greater stage on day 7, have a much higher chance of making a pregnancy than the ones that do not. A good quality day 7 IVP blastocyst or expanded blastocyst should have a defined inner cell mass and clear trophoblast cells (Figure 1). Shown in Figure 5 are a group of Day 7 IVP embryos at different stages of development and quality grades.

Data from our farm in Tables 4 to 6 present information for heifer and lactating cow recipients that supports our affirmation regarding embryo kinetics. All the embryos were transferred fresh unless noted. Table 4 shows the differences in pregnancy rates obtained in heifer and lactating cow recipients from grade 1 and 2 embryos (January 2017 to December 2019). We fitted a logistic model with recipient type, embryo stage, embryo grade, days from estrus, ET technicians, time of the year and interactions as fixed effects to the pregnancy variable. We ET embryos all year, and there was no significant difference in pregnancy rates for time of the year when we compared good to critical months. Embryos transferred 7 and 8 days after estrus have higher pregnancy rates. Lactating cows have higher pregnancy rates than heifer recipients probably because we are extra careful when selecting embryos for them. Only perfect blastocysts, with a defined inner cell mass (round) and very clear trophoblast cells, were selected (Figure 1). There was a significant decrease in pregnancy rates when grade 2 embryos were used and that is why we avoid using them in lactating cow recipients since these animals must get pregnant soon after calving to optimize economic returns. Experienced ET technicians have a significant positive impact on the results.

**Table 4:** Pregnancy rates for grade 1 and 2 embryos transferred fresh to heifer or lactating cow recipients (January 2017 to December 2019). (ET – Embryo transfer, Preg – Pregnancy rate).

**Embryo Grade/Recipient Type**	**Grade 1**		**Grade 2**		**Grades 1 and 2**	
	**ET**	**Preg**	**ET**	**Preg**	**ET**	**Preg**
Heifer Recipients	4204	51%	1599	34%	5803	46%
Lactating Cow Recipients	863	53%	80	31%	943	51%
Total	5067	51%	1679	34%	6746	47%

**Table 6:** Pregnancy rates for Grade 1 Day 7 IVP embryos transferred fresh to lactating cow recipients (January 2017 to December 2019) in two different times of the year. (ET – Embryo transfer, Preg – Pregnancy rate).

**Stage/Time of Year**	**Early Blastocysts or Blastocysts**						**Expanded or Hatched Blastocysts**					
	**Critical**		**Good**		**All Months**		**Critical**		**Good**		**All Months**	
**Estrus**	**ET**	**Preg**	**ET**	**Preg**	**ET**	**Preg**	**ET**	**Preg**	**ET**	**Preg**	**ET**	**Preg**
6	19	16%	9	44%	28	25%	110	41%	46	54%	156	45%
7	39	51%	64	47%	103	49%	232	56%	289	58%	521	57%
8	2	0%	6	50%	8	38%	25	56%	22	64%	47	60%
TOTAL	60	38%	79	47%	139	43%	367	52%	357	58%	724	55%

To better identify which factors affect pregnancy rates, we analyzed the grade 1 embryo pregnancy results separately. Since all our work is done in house, and we have natural heats on heifer recipients all week long, we can select some of the embryos on Day 6 of culture to be transferred. Table 5 summarizes the differences between grade 1 day 6 and day 7 IVP embryos transferred to heifer recipients at different days from estrus. Day 6 embryos have a higher pregnancy rate than Day 7 embryos. The embryos that do not reach blastocyst stage by day 6 or expanded blastocyst stage by day 7 had lower pregnancy rates, especially if transferred 6 days after estrus. The synchrony between embryo stage and days from estrus was not statistically different.

**Table 5:** Pregnancy rates for grade 1, Day 6 or 7 IVP embryos in different stages, transferred fresh to heifer recipients in different days of estrus (January 2017 to December 2019). (ET – Embryo transfer, Preg – Pregnancy rate).

**Day of Embryo/Stage**	**Day 6 Embryos**						**Day 7 Embryos**					
	**< Stage 6**		**>= Stage 6**		**Total**		**< Stage 7**		**>= Stage 7**		**Total**	
**Estrus**	**ET**	**Preg**	**ET**	**Preg**	**ET**	**Preg**	**ET**	**Preg**	**ET**	**Preg**	**ET**	**Preg**
6	10	50%	18	67%	28	61%	125	42%	250	34%	375	37%
7	137	61%	415	60%	552	60%	376	41%	790	50%	1166	47%
8	427	48%	750	61%	1177	56%	274	46%	538	50%	812	49%
TOTAL	574	51%	1183	60%	1757	57%	775	43%	1578	47%	2353	46%

Previously we tried to transfer Day 8 IVC blastocysts (they did not look viable on day 7, but reached the expanded blastocyst stage on Day 8), but we only had a 15% pregnancy rate (855 embryos from 2010 to 2019), so we decided to cease this practice.

Lactating cows can be used as recipients all year long with good pregnancy rates. Best results are achieved if a Day 7 expanded blastocyst or older is transferred at 7 or 8 days after estrus (Table 6).

In 2018, after major changes to our culture media were made, we started to freeze IVP embryos for direct transfer. So far, we have thawed 823 embryos at 7 and 8 days after estrus and have achieved a 48% pregnancy rate in both heifer and lactating cow recipients. We have noted that the pregnancy results with frozen-thawed embryos vary even more among ET technicians than fresh embryos, ranging from 36% to 60%.

We started with 25% pregnancy rates with fresh embryos in 2007 and now we can frequently attain pregnancy rates over 50% or higher. Lactating cows and heifers can be used as recipients all year around, with higher pregnancy rates when a grade 1, day 6 early blastocyst (or older) or day 7 expanded blastocyst (or older) is transferred by an experienced ET technician, 7 or 8 days after estrus.

## Conclusion

There are many factors involved to obtain a live calf from an IVP embryo. Attention to detail in every step of the process is crucial to success. The quality of the oocyte at the start of IVP is the key factor to determine the rest of the process. Due to the small number of oocytes recovered from Holsteins, we need to synchronize follicular wave emergence and stimulate with FSH to improve oocyte quality and consequently embryo production. Laboratory quality control and the use of high-quality supplies are essential to have less variability in production, facilitating the identification of other factors that might interfere with embryo production. Good quality embryos, selected at the right time and stage of development, transferred by an experienced ET technician, to well managed recipients, 7 or 8 days after estrus will result in high pregnancy rates. We keep track of data and maintain good records so we can analyze our results and take prompt action if necessary. The results and management recommendations presented here are based on the data collection, analysis and the actions taken to improve the IVP embryo production program at RuAnn Genetics. We always try to learn from good and poor results alike.
